# Body composition in adults with newly diagnosed type 2 diabetes: effects of metformin

**DOI:** 10.1186/s40200-014-0088-z

**Published:** 2014-08-21

**Authors:** Rokhsareh Aghili, Mojtaba Malek, Ameneh Ebrahim Valojerdi, Zahra Banazadeh, Laily Najafi, Mohammad Ebrahim Khamseh

**Affiliations:** Endocrine Research Center, Institute of Endocrinology and Metabolism, Iran University of Medical Sciences, Firouzgar alley, Valadi St., Behafarin St., Karimkhan Ave., Vali-asr Sq., Tehran, Iran; Lolagar hospital, Iran University of Medical Sciences, Tehran, Iran

**Keywords:** Diabetes Mellitus, Type 2, Insulin Sensitivity, Insulin Resistance, Sarcopenia, Dual Energy X-ray Absorptiometry

## Abstract

**Background:**

The aim of this study was to measure the body composition in adults with newly diagnosed type 2 diabetes mellitus and to explore the effect of metformin therapy on the various components of body composition, insulin sensitivity, and glucose homeostasis.

**Methods:**

This was an observational study consisted of 51 newly diagnosed people with type 2 diabetes on 1000 mg metformin twice daily for 6 months. The body composition of each subject was measured by dual energy X-ray absorptiometry at enrollment and 24 weeks after metformin mono-therapy. Sarcopenia was defined and compared based on the ratio of appendicular skeletal muscle and height squared, skeletal muscle index and residual methods.

Homeostasis model assessment-insulin resistance and Quantitative Insulin Sensitivity Check Index were used for estimating insulin sensitivity. The level of physical activity was assessed using self-administered International physical Activity questionnaire.

**Results:**

Forty one subjects (80.4%) completed the study. The mean age of the participants was 52.67 ± 10.43 years. Metformin treatment was associated with a significant decrease in total fat mass (−1.6 kg, P = 0.000). By week 24, the lean to fat ratio increased (P = 0.04) with men showing greater significant changes. Twenty percent of the female participants were detected to have sarcopenia.

In addition, there was a significant improvement of glucose homeostasis and insulin sensitivity.

**Conclusions:**

Metformin therapy results in significant improvement in body composition and insulin sensitivity of adults with newly diagnosed type 2 diabetes. Furthermore, sarcopenia begins in women with diabetes much earlier than expected as an age related phenomenon.

## Introduction

Type 2 diabetes is a prevalent disease with substantial morbidity and mortality [1,2]. It is now considered as a global health problem with serious economic burdens [3].

People with type 2 diabetes are usually overweight or obese [4]. Metformin, the most widely used biguanides for the treatment of type 2 diabetes mellitus [5], induces weight stabilization or small weight loss in adults with diabetes [6]. This might be due to reduction of insulin resistance and subsequent hyperinsulinemia [7,8]. It has also reported that in non-diabetic subjects with risk factors for type 2 diabetes mellitus, metformin modifies body composition by decreasing total fat content and increasing lean mass and water content [9].

Measuring body composition by dual energy X-ray absorptiometry (DEXA) is a noninvasive and valid method [10] that allows separation of the body mass into bone mass, fat mass (FM), and fat-free mass (FFM). It can also give estimation for the regional body composition [11].

Insulin resistance or insulin sensitivity were estimated using the homeostatic model assessment-insulin resistance (HOMA-IR) [12], and the Quantitative Insulin Sensitivity Check Index (QUICKI) methods [9].

The aim of this study was to investigate the body composition, the alterations happened by metformin, and their impact on insulin resistance, insulin sensitivity, and metabolic control in adults with newly diagnosed type 2 diabetes mellitus.

## Method and materials

### Study design

This was a 24-week, prospective, non-interventional, observational study of 51 newly diagnosed people with type 2 diabetes who had been initiated metformin to investigate the body composition, the alterations happened by metformin, and their impact on insulin resistance, insulin sensitivity, and metabolic control in routine clinical practice. G-power software was used to calculate the sample size. Assuming the mean and SD of fat weight [9] as one of the component of body composition that was important in this study, 38 patients will provide 90% power at a 2-sided with significance level of 0.05. Allowing for a 20% dropout rate, the number of subjects required for the study was 46 patients. Key eligibility requirements were fasting plasma glucose concentration of ≥7.0 mmol/L (≥126 mg/dl) or a 2-h plasma glucose concentration of 11.1 mmol/L (≥200 mg/dL) after 75 g glucose by mouth, or glycated hemoglobin (HbA1c) ≥6.5% (48 mmol/mol).

The diagnosis was confirmed by repeating one of the mentioned methods on a different day. Subjects with a diagnosis of cardiac, renal, pulmonary, endocrine and hepatic disease, or any other systematic intercurrent illness that might alter body composition were excluded. They were excluded if they had a GFR < 70 and/or an alanine aminotransferase (ALT) that exceeds 1.5 times the upper limit of normal. Those with prior treatment with oral glucose lowering drugs and/or insulin were also excluded.

### Patients

Fifty one subjects met the inclusion criteria and were enrolled in the study. Weight measurement was done using a calibrated digital scale (Seca gmbh & co. kg. Germany). A stadiometer (Seca gmbh & co. kg. Germany) calibrated before each measurement, was used for height measurement. Abdominal and hip circumferences were assessed by a trained nurse, using a cloth tape. The waist was defined at the midpoint between the highest point of the iliac crest and the lowest part of the costal margin in the midaxillary line, and the hip was measured at the level of the greater femoral trochanters. These measurements were used for calculating the body mass index (BMI), and the waist to hip ratio (WHR).

### Body composition and DXA assessment procedures

Body composition was assessed by Hologic whole body DEXA systems (QDR4500A, Hologic, Waltham, MA, USA; software version 8.21) A total body scan was performed at baseline and again at the end of the study. A standardized procedure for patient positioning and utilization of the QDR software was used. Total body FFM, FM, lean mass (LM), and percent fat were analyzed using Hologic version 8.21 software for tissue area assessment. Total body FFM was defined as lean soft tissue mass plus total body bone mineral content. Android FM was defined as adipose deposition around the abdomen; whereas, gynoid FM was adipose tissue accumulating around the hips. Appendicular skeletal muscle (ASM) was calculated as the sum of skeletal muscle mass in arms and legs. ASM/h^2^ was computed as the indicator of relative ASM. The scanner was calibrated daily against a spine calibration block and step phantom block supplied by the manufacturer. In addition, a whole body phantom was scanned weekly to assess any machine drift over time.

Skeletal muscle index (SMI) was analyzed based on the equation established by Janssen et al. (SMI = 100 × skeletal muscle mass/body mass) [13].

The predicted percentage of fat by sex, BMI, and age was calculated using the formulas proposed by Gallagher et al. [14].

Percentage of FM = 76.0 – 1097.8 (BMI^−1^) – 20.6 (Sex) + 0.053 (Age) + 95.0 (Asian) (BMI^−1^) – 0.044 (Asian) (Age) + 154 (Sex) (BMI^−1^) + 0.034 (Sex) (Age)

These formulas reported a correlation coefficient of about 0.90 and standard error of estimation of about 4%. Variables were defined as sex = 1 for male and 0 for female; Asian = 1 for Asian and 0 for other races [14].

### Physical activity assessment

At baseline, the level of physical activity was assessed with self-administered International Physical Activity Questionnaire (IPAQ). All of the participants were asked to have and maintain ≥ 30 min/day or 150 min per week of moderate-intensity such as walking or 75 min per week of vigorous aerobic physical activity or an equivalent combination of the two, based on recommendations of standards of medical care in diabetes 2012 [15]. Furthermore, each participant met a dietitian who administered a personalized isocaloric diet.

### Interventions and treatment

After an overnight fast of at least 8 hours, blood samples were obtained for measurement of fasting blood sugar (FBS) using a glucose analyzer (YSI 2700 Select, YSI, Inc., Yellow Springs, OH), fasting insulin (IMX assay, Abbott Laboratories, North Chicago, IL), and HbA1c using ion exchange chromatography (DS5 Analyzer, Drew Scientific limited, Cumbria, United Kingdom).

HOMA-IR index was used to estimate insulin resistance: HOMA-IR index = Fasting insulin (μU/mL) × Fasting glucose (mg/dL)/405.

Insulin sensitivity was determined by the quantitative insulin sensitivity check index (QUICKI) calculated as $\frac{1}{ \log \kern0.5em \mathrm{of}\kern0.5em \mathrm{fasting}\kern0.5em \mathrm{insulin}\kern0.5em \left(\upmu \mathrm{U}/\mathrm{mL}\right)\kern0.5em +\kern0.5em  \log \kern0.5em \mathrm{of}\kern0.5em \mathrm{fasting}\kern0.5em \mathrm{glucose}\kern0.5em \left(\mathrm{mg}/\mathrm{dL}\right)}$

Once baseline assessments were completed, metformin (Iran, Arya Pharmaceutical Company) was prescribed to all of the eligible subjects with a starting dose of 1000 mg/day.

After 12 weeks, the participants were reevaluated regarding their lifestyle and adherence to the medication. The metformin dose was titrated, if needed, based on HbA1c obtained after 12 weeks of starting treatment. The participants were followed by another 12 weeks.

### Sarcopenia classification

We compared the three methods used for identification of sarcopenia. Based on the definition by Baumgartner and colleagues, ASM/h^2^ was calculated as the ratio of ASM (kg) and height squared (m^2^) [16]. Participants were categorized as having sarcopenia based on ASM/h^2^ cut-points defined in previous studies (>2 *SD* below sex-specific means of normal reference population: 7.26 kg/m2 for men and 5.45 kg/m2 for women [16].

Considering SMI method, Class I sarcopenia was established in the participants whose SMI were within one to two SDs below the sex-specific mean of young adults. Class II sarcopenia was ascertained in the participants whose SMI were lower than two SDs below the sex-specific mean of young adults.

The residuals method is based on the regression model recommended by Newman et al. [17]. Linear regression equations using height (m) and FM (kg) to predict ASM were determined for male and female, respectively. The residuals of the regression were applied to identify participants whose ASM were lower or higher than the predicted. The 20th percentile of the distribution of residuals was used as the cutoff points for sarcopenia. Separate models were fit for men (ASM (kg) = −30.239 + 30.105 × height (m) + 0.141 × fat mass (kg)) and women (ASM (kg) = −15.407 + 17.595× height (m) + 0.162 × fat mass (kg).

The study was carried out at Endocrine Research Centre, Institute of Endocrinology and Metabolism. Ethical approval was obtained from the institutional review board of Institute of Endocrinology and Metabolism affiliated to Iran University of Medical Sciences (525MT/2011). All participants signed a written informed consent. Participants were free to withdraw at will at any time. If they withdrew, the data collected were used for analysis until the point when consent was withdrawn. The primary endpoints were changes in body composition and BMI by the end of 24 weeks. Secondary endpoints were insulin resistance, insulin sensitivity, HbA1c, fasting insulin, and fasting blood sugar.

### Statistical analysis

IBM SPSS for Windows Version 19 (IBM Corp., Armonk, NY, USA) was applied for the statistical analysis. The data were analyzed anonymously. Descriptive statistics (means and SDs) were used to describe key clinical and demographic characteristics. Normal assumptions were checked by looking at the Normal plot, or Frequency histogram with as well as Kolmogorov-Smirnov test. In case of normal distribution, we used parametric statistical test while we used non-parametric statistical tests in case of non-normal distribution. The relationship between age and ASM/h^2^, SMI, and total fat mass in men and women were illustrated by scatter plots and fit lines. Based on ASM/h^2^, SMI, and residuals methods, the prevalence of sarcopenia in men and women were calculated.

The comparison of the variables before and after the treatment was performed using the paired *t*-test or Wilcoxon. The relationships between variables were shown using Spearman’s Correlation coefficient. The ordinal variables were compared between two groups via the independent *t*-test or Mann–Whitney *U*-test. A P-value < 0.05 was considered statistically significant.

## Results

Of the 51 participants, a total of 41 subjects (80.4%) had good adherence to the study protocol and completed the 6-month study. Baseline anthropometric and laboratory indices did not significantly differ between those who finished the study and those who withdrew (data not shown). The mean age was 52.67 ± 10.43 years (range 26 – 71 years) and 59% were female. BMI was higher in women than in men. Table 1 illustrates descriptive baseline characteristics of the participants.

**Table 1:** **Descriptive baseline characteristics of the participants**

	**Female (n = 30)**	**Male (n = 21)**	**P-Value**
**Age (y)**	52.3 ± 10.58	53.2 ± 10.45	0.98
**Weight (kg)**	74.03 ± 10.82	81.09 ± 12	0.05
**BMI (kg/m** ^**2**^ **)**	30.06 ± 4.3	27.78 ± 3.27	0.04
**Waist (cm)**	101.83 ± 9.18	101.52 ± 7.6	0.90
**WHR**	0.95 ± 0.05	0.96 ± 0.04	0.26
**HbA1c (%)**	7.95 ± 2.19	8.45 ± 1.92	0.46
**HOMA-IR**	3.33 ± 1.53	3.95 ± 3.44	0.74
**QUICKI**	0.33 ± 0.02	0.33 ± 0.03	0.74

Independent sample *t*-test and Mann Whitney *U*-test were used.

BMI: Body Mass Index

WHR: Waist to Hip Ratio

HbA1c: Glycosylated Hemoglobin

HOMA-IR: Homeostatic Model Assessment-Insulin Resistance

QUICKI: Quantitative Insulin Sensitivity Check Index

Table 2 describes the changes of anthropometric variables by the end of the study. A significant sex difference was also noted (P = 0.000), with women showing a greater mean decrease in BMI (−0.62 Kg/m^2^) at 24 week with Metformin treatment than men (−0.58 Kg/m^2^). BMI was significantly related to total, gynoid, and android FM at baseline and the end of the study (P = 0.000). Waist circumference and WHR did not differ significantly by the end of the study in men and women. Waist circumference was mostly correlated with total FM (P = 0.000) and android FM (P = 0.000).

**Table 2:** **Changes in anthropometric variables by the end of the study**

**Characteristics**	**Female**		**P-value**	**Male**		**P-value**
	**Week 0**	**Week 24**		**Week 0**	**Week 24**	
**Weight (kg)**	74.03 ± 10.82	72.84 ± 10.76	0.00	81.09 ± 12	77.46 ± 15.07	0.09
**BMI (kg/m** ^**2**^ **)**	30.06 ± 4.3	29.44 ± 4.6	0.00	27.78 ± 3.27	27.18 ± 4.4	0.14
**Waist (cm)**	101.83 ± 9.18	99 ± 12.05	0.18	101.52 ± 7.6	99.46 ± 10.65	0.35
**WHR**	0.95 ± 0.05	0.95 ± 0.14	0.35	0.96 ± 0.04	0.95 ± 0.04	0.93

Paired *t*-test and Wilcoxon test was used.

BMI: Body Mass Index

WHR: Waist to Hip Ratio

The level of physical activity, classified as low, moderate, or high was assessed for all of the participants. It showed no significant difference by the end of the study, although, by week 24, more subjects reported higher level of physical activity (P = 0.082).

### Primary endpoints

At baseline and at the end of the study, a significant sex difference was shown with women having higher level of gynoid FM and total FM (P-values < 0.05).

By week 24, statistically significant reduction was observed in the total FM (−1.6 kg; 95% CI = −2.39 to 0.84; P =0.000) and percentage of FM (P = 0.000). There was also a significant increase in the proportion of lean/fat ratio in total study population (+0.10; 95% CI = 0.005 to 0.199; P = 0.04) that was more pronounced in men.

This was observed despite a reduction in LM (−1.07 kg, 95% CI = 1.78 to −0.35, P = 0.00) by the end of the study. ASM/h^2^ was also reduced by week 24 (−0.19 kg/m^2^; 95% CI = −0.35 to −0.029; P = 0.02).

SMI increased by week 24 (P = 0.02), with men showing a greater significant increase than women. Table 3 demonstrates body composition parameters at baseline and week 24.

**Table 3:** **Body composition parameters at baseline and 24 week by sex**

	**Female**			**Male**		
	**Week 0 (n = 30)**	**Week 24 (n = 27)**	**P-value**	**Week 0 (n = 21)**	**Week 24 (n = 14)**	**P-value**
Android fat mass (kg)	2.80 ± 0.77	2.63 ± 0.78	0.03	2.35 ± 0.79	2.04 ± 0.78	0.001
Gynoid fat mass (kg)	4.60 ± 1.02	4.42 ± 1.02	0.02	3.37 ± 0.83	3.11 ± 0.90	0.004
$\frac{\mathrm{Android}\kern0.5em \mathrm{fat}\kern0.5em \mathrm{mass}}{\mathrm{Gynoid}\kern0.5em \mathrm{fat}\kern0.5em \mathrm{mass}}$	0.61 ± 0.12	0.60 ± 0.13	0.45	0.70 ± 0.16	0.65 ± 0.12	0.015
Trunk/limb fat	1.02 ± 0.15	1.01 ± 0.15	0.47	1.21 ± 0.27	1.18 ± 0.24	0.25
Fat mass/h^2^ (kg/m^2^)	12.45 ± 3.1	11.88 ± 2.98	0.027	7.68 ± 2.1	7.11 ± 2.03	0.022
Appendicular lean mass/h^2^ (kg/m^2^)	7.49 ± 0.91	7.18 ± 1.03	0.006	8.75 ± 0.74	8.70 ± 0.92	0.38
Appendicular lean mass/W	0.24 ± 0.02	0.24 ± 0.02	0.29	0.31 ± 0.02	0.32 ± 0.02	0.69
SMI	0.03 ± 0.01	0.03 ± 0.01	0.6	0.038 ± 0.001	0.039 ± 0.001	0.005
Total fat mass (kg)	29.91 ± 6.91	28.68 ± 6.58	0.015	21.88 ± 5.87	20.12 ± 6.00	0.003
Percentage of Fat (%)	43.03 ± 5.35	41.54 ± 5.77	0.000	27.11 ± 3.61	25.82 ± 4.90	0.08
Total lean mass (kg)	41.95 ± 4.86	40.79 ± 5.12	0.014	53.6 ± 6.59	52.67 ± 7.60	0.17
Lean/Fat ratio	1.45 ± 0.31	1.48 ± 0.33	0.65	2.60 ± 0.69	2.8 ± 0.71	0.041

Paired *t*-test and Wilcoxon test was used.

h^2^: Height Squared

W: Weight

SMI: Skeletal Muscle Index

**Figure 1:**
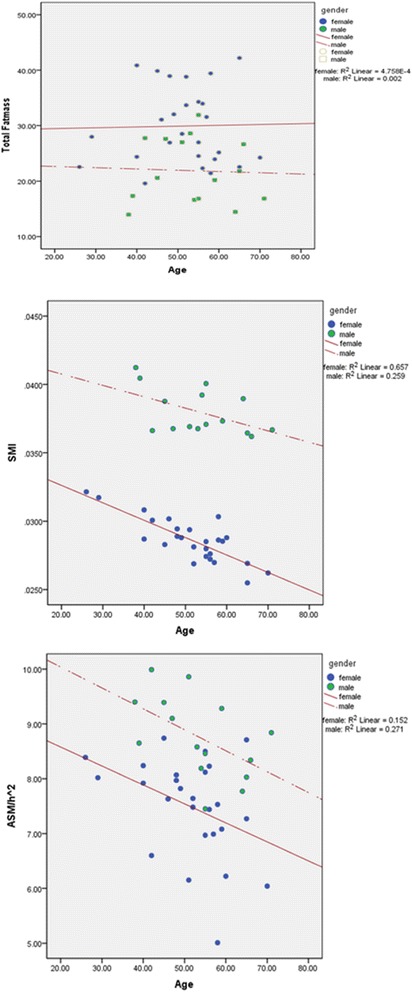
**The relationship between age and ASM/h** ^**2**^ **, SMI, and total FM in men and women.** ASM: Appendicular skeletal muscle. h^2^: height squared. SMI: Skeletal muscle index. FM: Fat Mass.

We also found an inverse relationship between ASM/h^2^, SMI, and age in both men and women. However, the total FM did not show similar trends (Figure 1).

**Figure 2:**
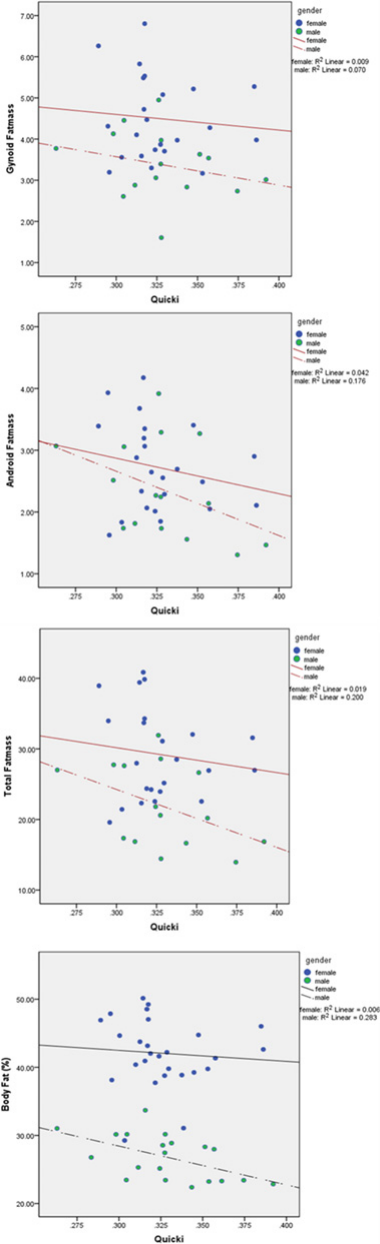
**The relationship between QUICKI (at the beginning of the study) and total FM, Percentage of body fat, android FM, and gynoid FM in men and women.** FM: Fat Mass.

Figure 2 shows the relationship between insulin sensitivity index (QUICKI) and total FM, percentage FM, android FM, and gynoid FM.

In both men and women insulin sensitivity increased as fat mass decreased.

### Secondary endpoints

Fasting blood sugar was improved significantly from 165.75 ± 60.75 mg/dL to 128.87 ± 40.1 (P = 0.0001). By week 24, statistically significant reductions in HbA1c (−1.34%; 95% CI = 1.99 to 0.68; P =0.000) and a significant increase in insulin sensitivity (QUICKI) (+0.01; 95% CI = 0.001 to 0.027; P = 0.03) was shown. However, ∆HOMA-IR was borderline statistically significant (P = 0.06).

Table 4 summarizes the changes in HbA1c, fasting serum insulin, insulin resistance (HOMA-IR), and sensitivity (QUICKI), by the end of the study.

**Table 4:** **Glycemic status, insulin resistance and sensitivity at baseline and week 24**

	**Week 0**	**Week 24**	**Mean difference ± SD**	**95% Confidence Interval**	**P-value**
HbA1c (%)	8.21±2.23	6.88±1.50	-1.34±2.05	(-1.99-0.68)	0.000
Fasting Insulin Level (μU/mL)	8.37±4.20	7.97±4.49	-0.4±5.49	(-1.45 2.26)	0.66
HOMA-IR	3.41±2.5	2.51±1.42	-0.90±2.79	(-1.84 0.45)	0.063
QUICKI	0.33±0.03	0.34±0.03	0.01±0.03	(0.001 0.027)	0.032

Paired *t*-test and Wilcoxon test was used.

HbA1c: Glycosylated Hemoglobin

HOMA-IR: Homeostatic Model Assessment-Insulin Resistance

QUICKI: Quantitative Insulin Sensitivity Check Index

### Exploratory endpoints

Based on ASM/h^2^ definition for sarcopenia, only one of the participants could be diagnosed as afflicted with sarcopenia. However, using the sarcopenia-residuals, 20% of the women were considered to have sarcopenia. Considering the low numbers of the male participants, we could not apply the formula for the men. The SMI method picked up a similar percentage of Class I (one SD below the mean value of young adults) sarcopenia for the women. We did not find Class II (two SDs below the mean value of young adults) sarcopenia in our study population.

The women with sarcopenia were older and their ASM/h^2^ was lower than non-sarcopenic women at baseline (P = 0.001) and week 24 (P = 0.004). However, there were not significant differences regarding metabolic indices and anthropometric variables at baseline and week 24 between sarcopenic and non-sarcopenic women.

## Discussion

Diabetes mellitus may be associated with increased risk of sarcopenia which can result in physical disability and metabolic disorders [18,19]. On the other hand, metformin therapy can improve the parameters of body composition and insulin dynamics in people who are at risk for type 2 diabetes [9]. The current study measured the body composition in adults with newly diagnosed type 2 diabetes mellitus and explored the effect of metformin therapy on various components of body composition, insulin sensitivity, and glucose homeostasis. The results showed significant changes in body composition, insulin sensitivity and glucose homeostasis after 24 weeks of treatment with metformin. The level of physical activity remained unchanged suggesting that the alterations happened in body composition of the participants were mainly due to the effect of the treatment.

We found a slight decrease in body weight in both sexes though the changes of BMI remained statistically significant only for the females probably due to predominance of the women participated in the study. The weight reducing effect of metformin has been demonstrated previously as a result of decreased food intake in a dose-dependent manner [6] It modifies body composition in subjects with type 2 diabetes by reducing total body and abdominal fat [6,20].

In a study conducted by Kim et al., it was shown that men with diabetes had decreased lean body mass and increased body fat mass, even with similar BMI compared with nondiabetic subjects. Based on the Framingham and the National Health and Nutrition Examination study III, the greater fat mass is associated with mobility limitations and fat mass may be more important for mobility than lean mass in older adults [21,22]. In addition, it has been shown that in women with sarcopenia, lean mass influenced mobility after accounting for body size and fat mass [23]. It was also shown that regardless of gender, people with diabetes had decreased SMI values in KSOS study while SMI values increased after 6-month metformin therapy in our study.

The insulin resistance level was reduced with metformin, but borderline statistical significance was observed. Furthermore, statistically significant change has been reported in insulin sensitivity as a result of decreased fasting plasma insulin and glucose level by metformin therapy. Compared to the glucose clamp technique, that is considered to be the gold standard method for directly measuring insulin sensitivity in vivo, QUICKI is a simple alternative with excellent reproducibility. The correlation between QUICKI and glucose clamp is significantly better than the correlation between HOMA-IR and clamp technique [24]. However, there was no statistical difference in insulin level by the end of the study. The increased insulin sensitivity observed by metformin therapy was in parallel to the improvement in glucose homeostasis and HbA1c reduction of 1.33% by the week 24. Our findings are in agreement with several previous studies [9,25].

Considering the results of KSOS, the prevalence of sarcopenia in patients with diabetes was significantly higher than non-diabetic subjects. In subjects older than 60 years, a significant difference in prevalence of sarcopenia between groups with and without diabetes was observed in both gender while in the middle-aged group (age 40 –59 years) this difference was observed only in women. The result of our study is consistent with KSOS that middle-aged women exhibited high prevalence of sarcopenia.

As shown in several previous studies, in general population men lose greater skeletal muscle mass with aging even with greater skeletal muscle mass compared to women [26,27]; however, women with diabetes are particularly considered high risk for loss of skeletal muscle mass [28]. These results suggest that type 2 diabetes may be an important risk factor for sarcopenia, particularly in women, considering its future impacts on quality of life, physical disability and mortality. Although we could not apply the definitions for men because of the low numbers of the male participants in our study, it seems this relationship in men could mainly be observed in elderly population.

There is still a lack of consensus on definition of sarcopenia [29]. Considering the three methods to define sarcopenia, different rates have been estimated for sarcopenia in our study. Based on the definition on ASM/h^2^, only one of the participants could be diagnosed as afflicted with sarcopenia. It has been reported that appendicular skeletal muscle, i.e. ASM/h^2^ would lead to a lower cut points for sarcopenia for the adults Asians [30] while based on the sarcopenia-residuals and the SMI definitions for sarcopenia out of five female participants, one had sarcopenia.

Taking the results of this study into consideration, further studies are needed to define a comprehensive method to define sarcopenia in people with type 2 diabetes considering body size, gender and ethnicity. Furthermore, it is needed to explore if female with diabetes are more prone to develop sarcopenia.

In conclusion, administration of metformin for six months had favorable effects on body composition, insulin sensitivity, and glucose homeostasis in adults with newly diagnosed type 2 diabetes and subsequently was expected to postpone the incidence of sarcopenia especially in women with type 2 diabetes who are at higher risk for loss of skeletal muscle mass.

### Limitations

Some limitations of this study include the limited number of the participants over the age of 60, small sample size, and lack of objective data about nutritional pattern of the participants.
